# Direct comparison shows that mRNA-based diagnostics incorporate information which cannot be learned directly from genomic mutations

**DOI:** 10.1186/s12859-020-3512-z

**Published:** 2020-05-19

**Authors:** Hersh D. Ravkin, Ofer Givton, David B. Geffen, Eitan Rubin

**Affiliations:** 1 Shraga Segal Department of Microbiology, Immunology and Genetics, Ben-Gurion University of the Negev, Beersheba, Israel; 2 Department of Oncology, Soroka University Medical Center, Faculty of Health Sciences, Ben-Gurion University of the Negev, Beersheba, Israel

**Keywords:** Gene expression, Genomics, Breast cancer recurrence, Oncology, Machine learning, Machine learning explainability, Data science

## Abstract

**Background:**

Compared to the many uses of DNA-level testing in clinical oncology, development of RNA-based diagnostics has been more limited. An exception to this trend is the growing use of mRNA-based methods in early-stage breast cancer. Although DNA and mRNA are used together in breast cancer research, the distinct contribution of mRNA beyond that of DNA in clinical challenges has not yet been directly assessed. We hypothesize that mRNA harbors prognostically useful information independently of genomic variation. To validate this, we use both genomic mutations and gene expression to predict five-year breast cancer recurrence in an integrated test model. This is accomplished first by comparing the feature importance of DNA and mRNA features in a model trained on both, and second, by evaluating the difference in performance of models trained on DNA and mRNA data separately.

**Results:**

We find that models trained on DNA and mRNA data give more weight to mRNA features than to DNA features, and models trained only on mRNA outperform models trained on DNA alone.

**Conclusions:**

The evaluation process presented here may serve as a framework for the interpretation of the relative contribution of individual molecular markers. It also suggests that mRNA has a distinct contribution in a diagnostic setting, beyond and independently of DNA mutation data.

## Background

Molecular testing has become an important tool in the clinical management of cancer patients and is often used for prediction, prognosis, and selection of therapy. Immunohistochemical staining for the HER2 receptor in breast cancer, PD-L1 receptor in lung cancer, and mismatch repair proteins in colon cancer are examples of protein-level molecular testing used to determine therapy [1–3]. DNA-level testing for gene mutations has also been widely adopted, mainly in the metastatic stage of disease, with many commercial next-generation sequencing gene panels available to individualize therapy [4]. Among early-stage cancers, DNA-level testing is used for HER2 gene copy evaluation in breast cancer and for evaluating microsatellite instability in colon cancer [2, 3]. Tumor mutation burden, as determined by DNA mutation evaluation, has also been evolving as a predictor of immunotherapy effectiveness in a range of metastatic malignancies [5]. Compared to the many uses of protein and DNA-level testing in clinical oncology, development of mRNA-based diagnostics has been more limited. Major reasons for this include the higher cost of mRNA testing and the greater ease of acquiring adequate DNA samples. Normalization and processing of mRNA, particularly when gathered from multiple laboratories, are also obstacles to the development and vetting of mRNA-based diagnostics on a larger scale. The somewhat roundabout method for testing HER2 overexpression is a good example of the preference for non-mRNA-expression-based methods: rather than testing HER2 expression directly, oncologists first examine protein expression and, if expression is equivocal, evaluate copy number aberrations (CNA), thus reaching an approximation of overexpression [3]. An exception to this trend is the growing use of mRNA-based methods in early-stage cancer, such as the 21-gene Recurrence Score Assay (Oncotype-DX™) and other commercially available platforms in breast cancer and a 12-gene expression test for prognosis in early-stage colon cancer [6–8]. Although mRNA is evaluated independently in these clinical applications, and DNA and mRNA are used together in breast cancer research [9] the distinct contribution of mRNA beyond that of DNA in clinical medicine has not yet been directly assessed. We hypothesize that mRNA harbors prognostically useful information independently of genomic variation. Here we describe a direct comparison of the relative prognostic utility of genomic mutations to that of gene expression, using both to predict five-year breast cancer recurrence in an integrated test model. We show that such a model gives more weight to mRNA features than to DNA features. The evaluation process presented here may serve as a framework for the interpretation of the relative contribution of individual molecular markers. It also suggests that mRNA has a distinct contribution in a diagnostic setting, beyond and independently of DNA mutation data.

## Results

### Overview

The goal of this work was to directly test the hypothesis that mRNA expression data contains prognostically-relevant information independently of mutation profile. Two predictions are tested in this work: (1) models developed on mutation profiles alone will be less successful than models developed on mRNA levels and (2) methods that calculate relative multivariate feature importance will attribute higher predictive value to mRNA features than to DNA features in models in which both are used. To test these predictions, we used the METABRIC dataset, the only dataset of its size that contains both mutation and mRNA profiles [9–11]. The input matrix we used included mutations data for all the 173 genes analyzed in METABRIC as well as 17,299 mRNA microarray-based measurements which were retained after pre-processing and selected clinical features (e.g. ER Status). Predictive models of 5-year recurrence were trained using XGBoost (eXtreme Gradient Boosting, Version 0.80), a powerful decision tree-based ensemble machine learning algorithm [12]. Feature importance was scored using the SHAP (SHapley Additive exPlanations) package (Version 0.25.1), the most robust approach currently available for explaining machine learning outputs [13]. For general pipeline development and validation, scikit-learn (Version 0.20.0) was used [14].

### Model evaluation

**Fig. 1:**
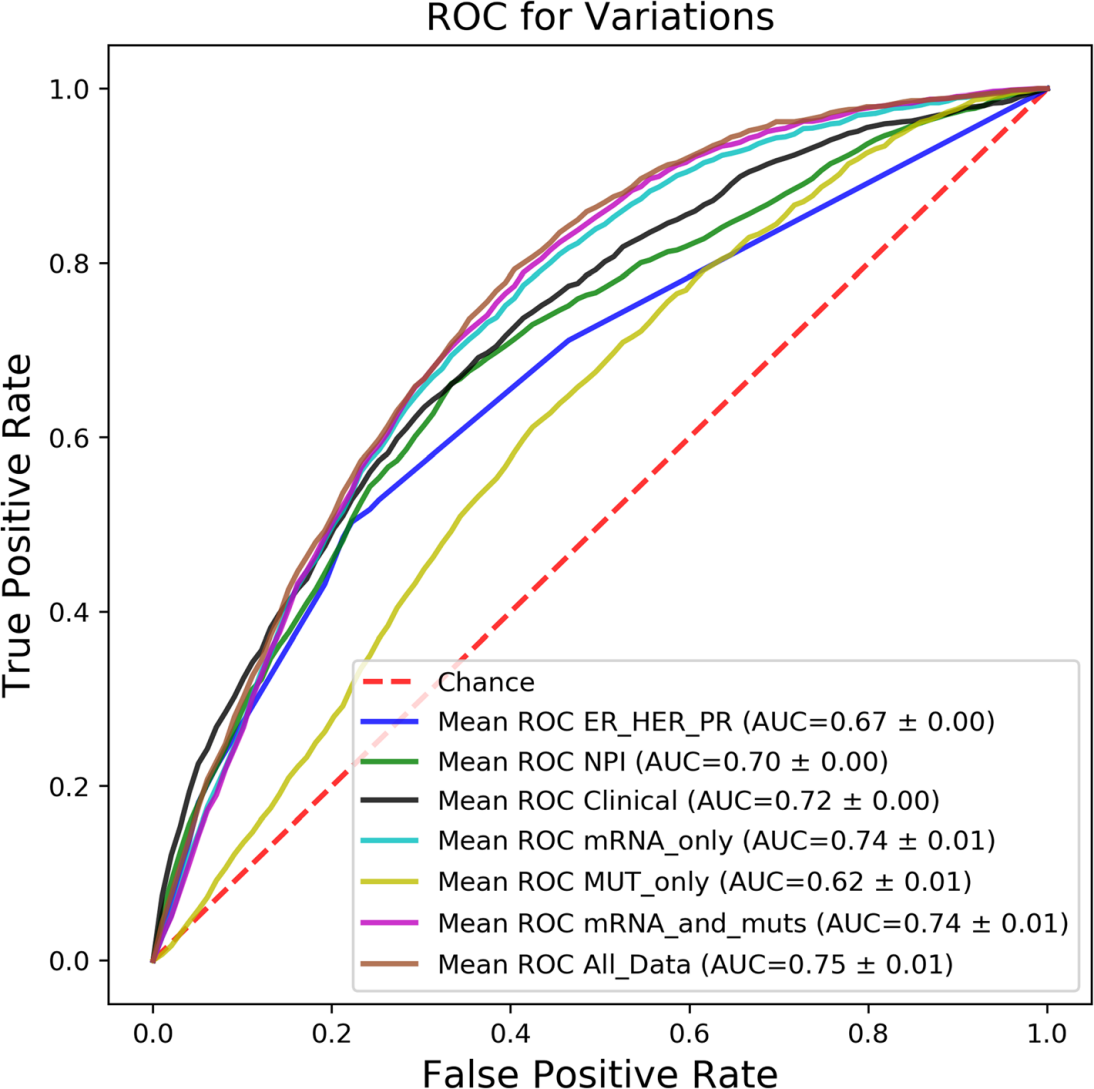
ROC Plot showing the performance of the different models, each trained on different subsets of the full data

To avoid issues of normalization between mRNA data collected from the different laboratories in METABRIC, the largest cohort was chosen as the training-testing set, while the 4 remaining cohorts were kept for hold-out validation. Ten-fold cross-validation was used on the training-testing set and performance of the model from each of the folds was also evaluated on the hold-out data. All results presented here reflect the average performance of the cross-validation models when predicting on the hold-out data. The resulting XGBoost models performed better than random when predicting five-year breast cancer recurrence in the validation data (see Fig. 1). The model trained only on mutation data was the least successful and it too had an AUC greater than 0.5 when predicting on the validation data. Besides establishing the ability of XGBoost to train transferrable predictive models with the data used here, preliminary comparison of the different models’ AUC alone supports our hypothesis: models based on mutations alone do not perform as well as models based on mRNA alone (AUC = 0.74 compared to 0.62). Moreover, the Matthew’s Correlation Coefficient (MCC) for each fold was significantly higher in models trained on mRNA features only compared to models trained only on DNA (*p* = 4.5 × 10^− 10^, paired t-test; t-statistic = − 28.1). The same trend of mRNA surpassing mutations in predicting recurrence was observed when using logistic regression (data not shown).

### Direct comparison of mRNA and mutation features

**Fig. 2:**
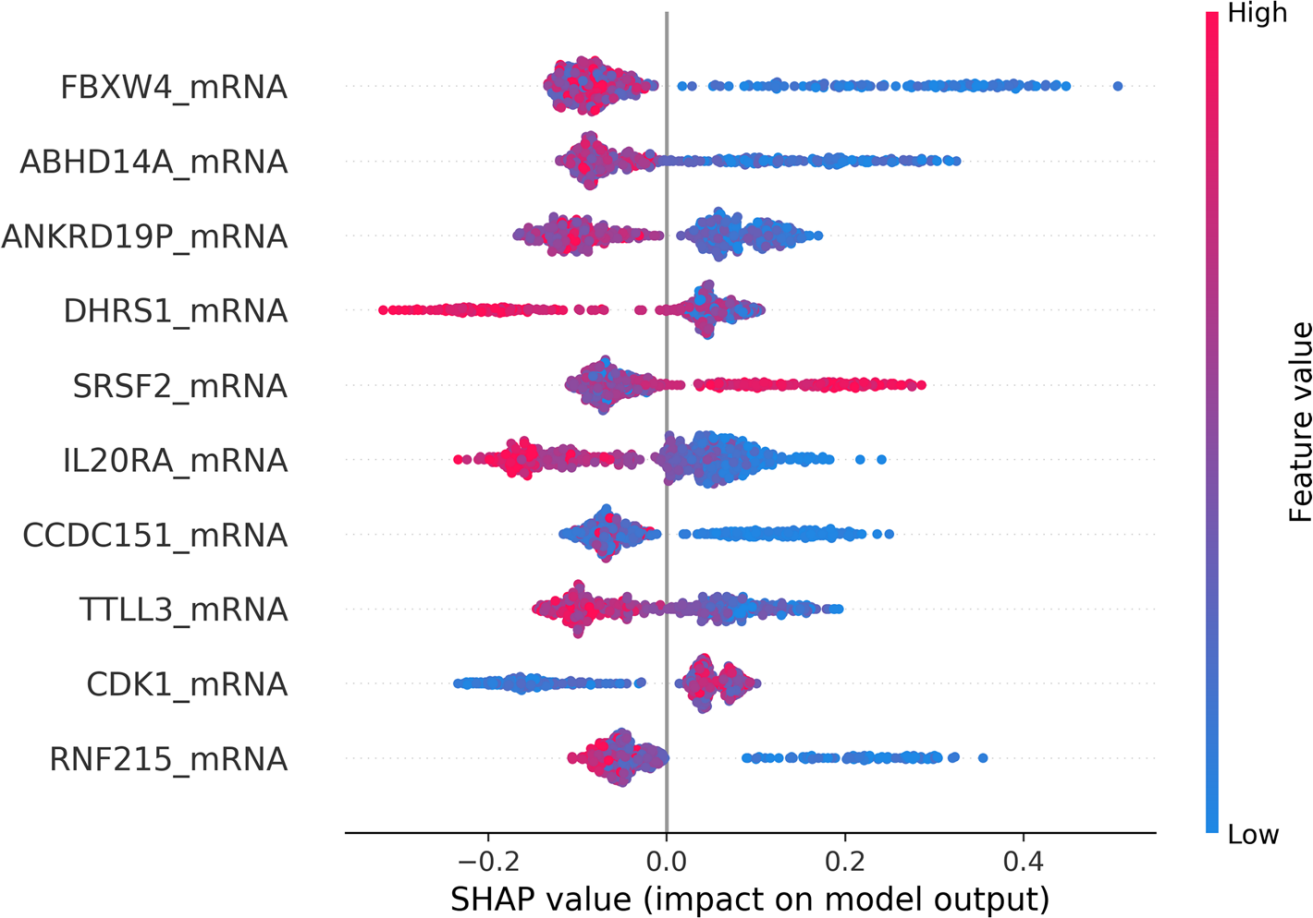
SHAP plot of the 10 most prognostic features. This graph shows SHAP values averaged across all the folds when the model was trained on mRNA and mutation data together. Each point on the graph represents a sample from the validation data. The color of each point represents the actual value of that feature. Greater absolute value on the x-axis indicates higher impact on prediction

Multivariate feature importance (SHAP value) was calculated for each feature and the SHAP value of each feature was averaged across all folds to generate one value per feature. The 10 features with the highest SHAP score when trained on the mRNA and mutation data together are presented in Fig. 2. The features with the highest SHAP values were relatively consistent across all folds and mRNA features ranked in the top hundred in every fold in which they were included. Although some mutation data was retained in some of the folds when the model was trained on the mutation and mRNA data together, mutation data was never stable across the 10 folds and the mean SHAP value of every mutation feature was zero. The uniformly colored streaks on either side of the y-axis are common throughout the highest performing features. These streaks, given the mixed-color swarms on the other side of the y-axis, suggest interaction with other features (e.g. FBXW4_mRNA). Progressive change in color for a feature suggests a linear relationship of feature value with impact on model output (e.g. TTLL3 mRNA). A gene analysis was performed to seek commonality within the top features and known cancer pathways. The analysis failed to identify significant enrichment using hypergeometric tests in known gene sets (data not shown).

## Discussion

Results of this work support the hypothesis that mRNA levels carry clinically important information which cannot be learned directly from mutation data. The relative power of mRNA and DNA features in predicting 5-year breast cancer recurrence was directly compared. SHAP values were used to quantify the relative importance of DNA and mRNA features in our model, and indicated that only mRNA features were consistently predictive of recurrence (Fig. 2). As expected, models that are built on mRNA alone are very similar in AUC to models built using mRNA and DNA, but are much more accurate than models based on DNA alone. Moreover, models that include mRNA outperform models based only on clinical information (including information about clinical subtyping). We speculate that the lack of significant commonality within top-scoring features and known cancer pathways in gene analysis may stem from differences between the biological processes involved in cancer development and recurrence. Because cancer development is better understood, known gene sets may possibly be biased against recurrence-associated pathways.

It is well established that breast cancer recurrence rates differ between the main clinical subtypes. Research literature further stratifies those subtypes into even more distinct subtypes based on integrative clusters of molecular markers which are associated with even more specific recurrence profiles [9–11]. The behavior of the model can be understood as associating samples with distinct subtypes, and thus predicting recurrence based on recurrence rates for that subtype. This could also explain why models trained on clinical variables alone perform relatively well: both models trained on molecular data and those trained on clinical data may be predicting largely based on belonging to a particular subtype. The improved performance of models trained on mRNA data may thus reflect greater success of mRNA, compared to DNA, in associating samples with the correct subtype. Alternatively, mRNA levels may themselves be directly indicative of recurrence likelihood. The ability of the 21-gene assay (used in ER+ breast cancer only) to predict recurrence within the ER+ subtype more accurately than any DNA-based assay supports this explanation. It suggests that the assay can identify pathway alterations on a more detailed level than would be possible by simply associating with subtype. There are many genomic alterations which can lead to the activation of a single downstream pathway and thus cause a characteristic expression pattern. Such a pattern is more easily identified by directly examining gene expression, further explaining why models trained on mRNA may be better able to indicate that a tumor has recurrence-driving mechanisms in place.

In either case, the likely reason why mRNA levels are more informative than genomic variation in predicting recurrence is due to their larger dynamic range and higher-level indication of phenotype. Poorer performance by models trained on genomic variation alone likewise reinforces one of the central issues in machine learning with genomic data: there are too many intermediate steps between driver mutations and disease phenotype to feasibly incorporate most genomic changes directly into a predictive model [15]. Understanding the connection between specific genomic changes and disease phenotype is essential to better understanding the progression of breast cancer. However, to usefully incorporate genomic data into predictive models trained on datasets of comparable size to those used here, a greater level of abstraction is needed. Future directions for research may include attaining such abstraction by examining genomic variants as members in molecular pathways, rather than as lone markers.

Besides increasing abstraction, feature selection based on prior knowledge can also reduce the amount of data needed to train machine learning models. The comparison of 17,299 mRNA features with only 173 genes may be seen as a potential area for concern in the planning of this project. Rather than being a setback, however, we see this as a good example of incorporating prior knowledge into a machine learning pipeline. The selection of these genes for inclusion in METABRIC was based on their frequency of harboring mutations and being targeted for homozygous deletions [10]. It is likely that irrelevant genes would not have been retained after feature selection in any case. The inclusion of the logistic regression addressed another potential concern: because we utilized a tree-based model, one-hot encoding of mutations may have caused them a performance disadvantage relative to numerically encoded mRNA (see Methods for a brief explanation of one-hot encoding). The results of the logistic regression, a non-tree-based method, alleviate this concern.

The ability of the model to predict accurately on unseen data despite being trained on and predicting across various cancer subtypes supports the feasibility of using machine learning to build comprehensive models to predict breast cancer recurrence across all subtypes. The success of examining multivariate feature importance in our model suggests that as more genomic and transcriptomic data becomes available, these same analyses could be used with more comprehensive models to uncover specific cancer mechanisms. For instance, examining which expression markers interact more significantly with ER+/− breast cancer could help elucidate the different pathophysiology of the respective subtypes. Taken together, increasing abstraction and examining the multivariate relative contribution of various prognostic factors are key to interpreting more complex models.

The results presented here can also be used for other purposes. Our successful use of SHAP to examine the relative importance of features reinforces the use of this relatively new technology in other clinical areas [16] and suggests its potential for use with other feature types (e.g. pathological images, protein levels) in the medical field. While beyond the scope of this work, it would be interesting to test SHAP in comparing other diagnostic feature types in biomedical research, as well as in characterizing the exact impact of each features on model outcomes.

The potential use of mRNA profiling in other precision oncology challenges has recently been further validated in a clinical trial, although other uses in clinical care have not yet been established [17]. The growing use of mRNA in diagnostics requires expanding the characterization of how mRNA diagnostics function differently from other molecular diagnostic platforms, both for gaining deeper insight into breast cancer transcriptomics and for development of further mRNA diagnostics and greater understanding of the type of clinical questions which they can answer. Despite the commercial availability of mRNA-based diagnostics for other cancers [18, 19], more advanced understanding of mRNA diagnostics is likely to stem from further research into their use in breast cancer, which is the most strongly validated and documented. Considering the results presented here, further investigation of breast cancer recurrence with the expanded information afforded by mRNA profiling indeed seems worthwhile.

## Conclusions

We conclude that mRNA can reveal clinically important information which cannot be learned directly from mutation data. The use of DNA- and mRNA-based diagnostics in clinical practice is constantly growing, and greater understanding of their mutual and exclusive capabilities will be required for development of new diagnostic methods. The framework presented here is thus relevant beyond the field of breast cancer recurrence and can be used to address the question more generally.

## Methods

### Data sources

Clinical and molecular data were taken from the METABRIC study. The data is described in Curtis, C., Shah, S.P., et al. 2012. Briefly, it includes over 2000 samples collected from various tumor banks. For each of these samples, the mutation data consists of somatic mutations in 173 sequenced genes and was provided as a list of all mutations found in any of the patients. The mRNA expression data for these patients was collected on Illumina Human v3 microarrays. Mutation, mRNA, and clinical data were downloaded from cBioPortal (https://www.cbioportal.org/study/summary?id=brca_metabric, access date: 19.02.2019) [20, 21]. The Z-score normalized expression values available from cBioPortal were used. Additional matched recurrence data was downloaded separately from a subsequent study on the same patients and is described in Rueda, O.M., Sammut, S., et al. 2019. Data from the various sources was matched by patient ID.

### Pre-processing of mRNA and clinical data

Pre-processing steps for the clinical and mRNA data included: (1) removal of repeating features (*n* = 285); (2) removal of features in which all values were missing (*n* = 959); (3) imputation of missing values (9 features in the mRNA data and 8 features in the clinical data had remaining missing values); and (4) one-hot encoding of nominal features (ER, HER2, and PR status and breast tumor laterality, histological type, and recurrence history). In this case, one-hot encoding refers to transforming a categorical feature into a binary one, e.g. recoding the category “Gender” into two binary features, “Male” and “Female.” After the above steps, information on 17,299 mRNA features was retained for use in the eventual input matrix. All clinical data pertaining to treatment regimen was removed as the samples in the dataset had received homogenous treatments within clinically relevant groupings. Ages of the participants were likewise removed to reduce possible bias from deaths not related to disease.

### Pre-processing of mutation data

Mutation load (mutated genes per patient) was calculated for each patient as the number of mutations (of any type) and included as a separate feature. After mutation load was calculated, silent mutations (*n* = 4063) were removed from subsequent analysis leaving only mutations likely to affect mRNA levels and/or protein function. Dimensions of the mutation data were reduced by categorizing the remaining mutation variants described in the original data into three levels, according to mutation severity:

- Level 1: Intron (*n* = 49), translation start site (*n* = 5), nonstop (*n* = 5), 3’UTR (*n* = 4), 5’flank (*n* = 3), 3’flank (*n* = 3)
- Level 2: Missense (*n* = 10,165), in-frame deletions (*n* = 250), in-frame insertions (*n* = 61)
- Level 3: Nonsense (*n* = 836), frame-shift deletions (*n* = 825), frame-shift insertions (*n* = 477), splice site (*n* = 402), splice region (*n* = 124)

One-hot encoding was subsequently applied to each level where applicable. In patients with more than one mutation in the same gene, only the most severe mutation was retained. The resultant matrix contained 344 columns describing the applicable mutation levels of the 173 genes analyzed in the study.

### Pre-processing of combined data

Finally, recurrence was used as the target variable, defined as distant recurrence in the span of 5 years. Patients with less than 5 years of follow-up, either because they dropped out of the study or because of disease-unrelated death, were removed from the analysis. The largest available cohort (*n* = 516) was chosen for training-testing with 10-fold cross validation, while the remaining four cohorts were used as a hold-out validation dataset. Class imbalance in the training-testing cohort was reduced to a 2:1 ratio by subsampling instances of the majority class (there were significantly more patients who survived for more than 5 years). Data was stratified to contain equal instances of each target variable class across 10 folds.

### Model development pipeline

The XGBoost algorithm (Version 0.80) was used with the scikit-learn API (Version 0.20.0) for generating the predictive model [12, 14]. Feature selection for the main XGBoost models was performed with scikit-learn’s SelectFromModel meta-transformer, which itself was set to use the XGBoost algorithm for ranking the highest-performing features. Six hundred features were retained after the selection (this number was arbitrarily chosen to allow subsequent analysis of relative importance). Feature selection was performed for each fold individually to prevent test leakage. XGBoost was run with default parameters with the following changes: max_depth = 7, learning_rate = 0.05, colsample_bytree = 0.3, n_jobs = − 1. Additionally, a logistic regression analysis was performed (using scikit-learn’s LogisticRegression function) in each fold. The features entered into the regression underwent their own feature selection (also set to retain the 600 best performing features) using scikit-learn’s SelectKBest function. The model was run in a loop and trained on one of the following seven variations of the data in each round: ER/HER2/PR status only, Nottingham Prognostic Index (NPI) only, all of the included clinical variables, mRNA expression only, mutation data only, mRNA and mutation data together, and all of the data (molecular data and clinical data). All the results shown reflect the average performance of the cross-validation models when predicting on the validation hold-out set.

### Evaluation of feature contribution

Contribution of each feature to the model was evaluated with the SHAP package (Version 0.25.1) [13], chosen for its robust estimation of multivariate feature importance. Assessment of relative importance of the various features was based on the average SHAP values of each feature across the validation folds.

Availability: The code, input matrixes, and configuration file are provided as supplementary material.

## Supplementary information

**Additional file 1.**

## Data Availability

Data sharing is not applicable to this article as no datasets were generated during the current study. For instruction on how to obtain the original datasets analyzed during the current study see Methods or contact the corresponding author. For instructions on how to obtain the software used in this work, see Methods.

## Abbreviations

HER2: Human Epidermal Growth Factor Receptor 2
PDL-1: Programmed Death-Ligand 1
DNA: Deoxyribonucleic Acid
mRNA: Messenger Ribonucleic Acid
CNA: Copy Number Aberrations
METABRIC: Molecular Taxonomy of Breast Cancer International Consortium
XGBoost: eXtreme Gradient Boosting
SHAP: SHapley Additive exPlanations
AUC: Area Under the Curve
ROC: Receiver Operating Characteristic
MCC: Matthew’s Correlation Coefficient
ER: Estrogen Receptor
PR: Progesterone Receptor
API: Application Programming Interface
NPI: Nottingham Prognostic Index

## References

[1] Ettinger DS, Aisner DL, Wood DE, Akerley W, Bauman J, Chang JY (2018). NCCN guidelines insights: non–small cell lung Cancer, version 5.2018. J Natl Compr Canc Netw.

[2] Taieb J, Shi Q, Pederson L, Alberts S, Wolmark N, Van Cutsem E (2019). Prognosis of microsatellite instability and/or mismatch repair deficiency stage III colon cancer patients after disease recurrence following adjuvant treatment: results of an ACCENT pooled analysis of seven studies. Ann Oncol.

[3] Wolff AC, Hammond ME, Allison KH, Harvey BE, Mangu PB, Bartlett JMS (2018). Human epidermal growth factor receptor 2 testing in breast Cancer: American Society of Clinical Oncology/College of American Pathologists Clinical Practice Guideline Focused Update. Arch Pathol Lab Med.

[4] Zeng J, Johnson A, Shufean MA, Kahle M, Yang D, Woodman SE (2019). Operationalization of next-generation sequencing and decision support for precision oncology. JCO Clin Cancer Inform.

[5] Budczies J, Allgäuer M, Litchfield K, Rempel E, Christopoulos P, Kazdal D (2019). Optimizing panel-based tumor mutational burden (TMB) measurement. Ann Oncol.

[6] Geffen DB (2018). Should decisions on adding adjuvant chemotherapy in early-stage ER-positive breast cancer be based on gene expression testing or clinicopathologic factors or both?. Ann Oncol.

[7] Sparano JA, Gray RJ, Makower DF, Pritchard KI, Albain KS, Hayes DF (2015). Prospective validation of a 21-gene expression assay in breast Cancer. N Engl J Med.

[8] You YN, Rustin RB, Sullivan JD (2015). Oncotype DX® colon cancer assay for prediction of recurrence risk in patients with stage II and III colon cancer: a review of the evidence. Surg Oncol.

[9] Curtis C, Shah SP, Chin S, Turashvili G, Rueda OM, Dunning MJ (2012). The genomic and transcriptomic architecture of 2,000 breast tumours reveals novel subgroups. Nature.

[10] Pereira B, Chin S, Rueda OM, Vollan HM, Provenzano E, Bardwell HA (2016). The somatic mutation profiles of 2,433 breast cancers refine their genomic and transcriptomic landscapes. Nat Commun.

[11] Rueda OM, Sammut S, Seoane JA, Chin S, Caswell-Jin J, Callari M (2019). Dynamics of breast-cancer relapse reveal late-recurring ER-positive genomic subgroups. Nature.

[12] Chen T, Guestrin C (2016). XGBoost: A Scalable Tree Boosting System. Proceedings of the 22nd ACM SIGKDD International Conference on Knowledge Discovery and Data Mining.

[13] Lundberg SM, Lee S (2017). A unified approach to interpreting model predictions. Advances in Neural Information Processing Systems.

[14] Pedregosa F, Varoquaux G, Gramfort A, Michel V, Thirion B, Grisel O, Blondel M, Prettenhofer P, Weiss R, Dubourg V, Vanderplas J, Passos A, Cournapeau D, Brucher M, Perrot M, Duchesnay É (2011). Scikit-learn: machine learning in python. J Mach Learn Res.

[15] Leung MKK, Delong A, Alipanahi B, Frey BJ (2016). Machine learning in genomic medicine: a review of computational problems and data sets. Proc IEEE.

[16] Lundberg SM, Nair B, Vavilala MS, Horibe M, Eisses MJ, Adams T (2018). Explainable machine-learning predictions for the prevention of hypoxaemia during surgery. Nat Biomed Eng.

[17] Rodon J, Soria J, Berger R, Miller WH, Rubin E, Kugel A (2019). Genomic and transcriptomic profiling expands precision cancer medicine: the WINTHER trial. Nat Med.

[18] Renfro LA, Zhang N, Lopatin M, Chao C, Alberts SR (2017). Prospective evaluation of a 12-gene assay on patient treatment decisions and physician confidence in mismatch repair proficient stage IIA Colon Cancer. Clin Colorectal Cancer.

[19] Eggener S, Karsh LI, Richardson T, Shindel AW, Lu R, Rosenberg S (2019). A 17-gene panel for prediction of adverse prostate Cancer pathologic features: prospective clinical validation and utility. Urology.

[20] Gao J, Aksoy BA, Dogrusoz U, Dresdner G, Gross B, Sumer SO (2013). Integrative analysis of complex cancer genomics and clinical profiles using the cBioPortal. Sci Signal.

[21] Cerami E, Gao J, Dogrusoz U, Gross BE, Sumer SO, Aksoy BA (2012). The cBio Cancer Genomics Portal: An Open Platform for Exploring Multidimensional Cancer Genomics Data. Cancer Discov.

